# Supplementary material for: Evaluation of Cooked Rice for Eating Quality and Its Components in Geng Rice
Source: Foods. 2023 Aug 30;12(17):3267. doi: 10.3390/foods12173267 (PMC10486766; doi:10.3390/foods12173267)
Supplement: Supplementary file 1 [file foods-12-03267-s001.zip › Supplemental Figures.pdf]

Supplemental figure:

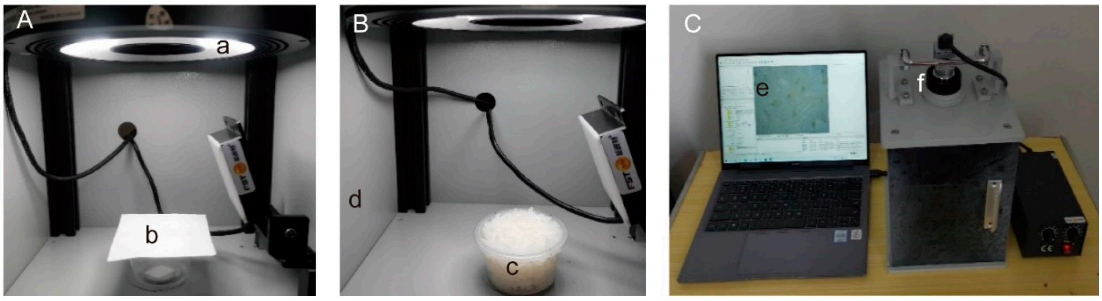

Figure S1. The process used for imaging cooked rice.

Color calibration (A), sample preparation (B), and imaging (C). The lower-case letters designate the dome lamp (a), A4 white paper (b), cooked rice in the container (c), the gray acrylic sheets(d), images of cooked rice (e), and the telecentric lens (f).

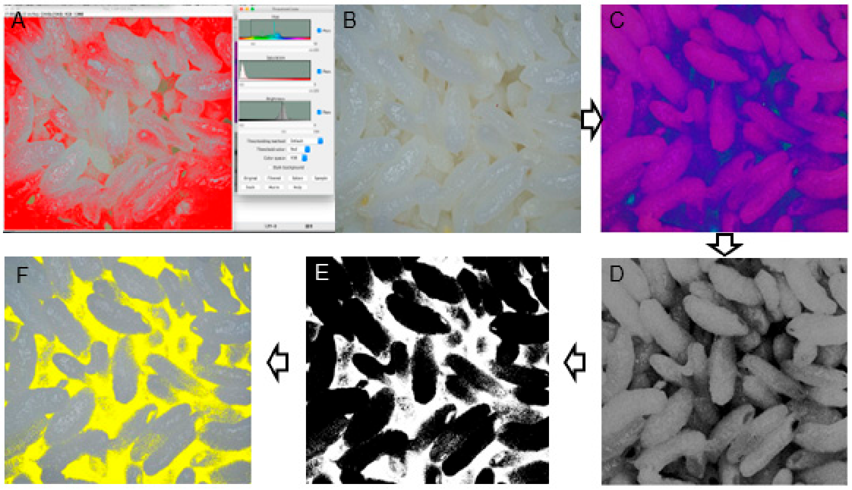

Figure S2. The process used to extract the yellow area in the cooked rice images.

Image in ImageJ (A), RGB image in matlab (B), the HSV image (C), extracting the image hue channel (D), the binary image (E), displaying the pixels for the yellow area (F).

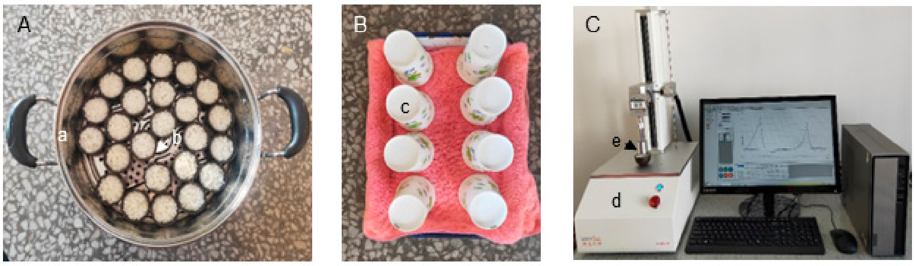

Figure S3. The process of measuring viscosity in cooked rice.

Cooked rice in the steamer (A), cooling the cooked rice (B), measuring the viscosity parameters of the cooked rice using the Texture Analyzer (C). Steamer (a), Zisha cup (b), paper cup (c), Texture Analyzer (d), probe (e).
