# Supplementary material for: Evaluation of Cooked Rice for Eating Quality and Its Components in Geng Rice
Source: Foods. 2023 Aug 30;12(17):3267. doi: 10.3390/foods12173267 (PMC10486766; doi:10.3390/foods12173267)
Supplement: Supplementary file 1 [file foods-12-03267-s001.zip › Supplemental Tables.pdf]

Supplementary Tables:

Table S2 Correlation analysis between viscosity and parameters of color models CIEL*a*b*and HSI (2020)

	L	a*	b*	H	S	I	Appearance
L	1						
a*	-0.27**	1					
b*	0.09	-0.11	1				
H	-0.06	-0.03	-0.98**	1			
S	-0.08	-0.35**	-0.59**	0.62**	1		
I	1.00**	-0.25**	0.04	-0.01	-0.06	1	
Appearance	0.10	0.08	0.12	-0.13	-0.14	0.10	1

** Indicates significance at $P < 0.01$.

Table S3 Correlation analysis between viscosity and parameters of Texture Analyzer for cooked rice (2020)

	TPA-hardness	TPA-adhesiveness	TPA-springiness	TPA-chewiness	TPA-gumminess	TPA-cohesiveness	TPA-resilience	Viscosity
TPA-hardness	1							
TPA-adhesiveness	0.22**	1						
TPA-springiness	0.15*	0.63**	1					
TPA-chewiness	0.72**	0.55**	0.75**	1				
TPA-gumminess	0.93**	0.34**	0.37**	0.89**	1			
TPA-cohesiveness	-0.02	0.41**	0.65**	0.57**	0.34**	1		
TPA-resilience	0.15*	-0.03	0.16*	0.37**	0.39**	0.72**	1	
Viscosity	-0.02	0.42**	0.31**	0.23**	0.10	0.32**	0.15*	1

* Indicates significance at $P < 0.05$; ** Indicates significance at $P < 0.01$.

TPA: Parameters measured by the Texture Analyzer.

Table S4 Correlation analysis between viscosity and parameters of Texture Analyzer for cooked rice (2021)

	TPA-hardness	TPA-adhesiveness	TPA-springiness	TPA-chewiness	TPA-gumminess	TPA-cohesiveness	TPA-resilience	Viscosity
TPA-hardness	1							
TPA-adhesiveness	-0.18*	1						
TPA-springiness	0.053	0.59**	1					
TPA-chewiness	0.74**	0.09	0.53**	1				
TPA-gumminess	0.84**	-0.19**	0.15*	0.91**	1			
TPA-cohesiveness	0.02	-0.09	0.31**	0.40**	0.30**	1		
TPA-resilience	0.31**	-0.41**	-0.01	0.48**	0.56**	0.82**	1	
Viscosity	-0.28**	0.58**	0.25**	-0.16*	-0.32**	-0.09	-0.31*	1

* Indicates significance at $P < 0.05$; ** Indicate significance at $P < 0.01$.

TPA: Parameters measured by the Texture Analyzer.
